# Editorial: Endophytic fungi: from plant occupants to agricultural allies

**DOI:** 10.3389/fmicb.2026.1982175

**Published:** 2026-09-30

**Authors:** Rosario Nicoletti, Jolanta Jaroszuk-Ściseł, Beata Zimowska

**Affiliations:** 1 Research Centre for Olive, Fruit and Citrus Crops, Council for Agricultural Research and Economics, Caserta, Italy; 2 Department of Industrial and Environmental Microbiology, Maria Curie-Skłodowska University, Lublin, Poland; 3 Department of Plant Protection, University of Life Sciences, Lublin, Poland

**Keywords:** antagonism, defensive mutualism, endophytic fungi, plant growth promotion, secondary metabolites

For decades, endophytic fungi were regarded as inconspicuous plant inhabitants, organisms capable of colonizing living tissues without causing visible symptoms. Recent research has profoundly changed this perception, and endophytes are now recognized as key components of plant microbiomes, able to promote growth, improve stress tolerance, protect against pathogens, and contribute to more sustainable agricultural systems. Increasing our knowledge on their occurrence and properties is essential for facilitating their transition from pure ecological curiosity to a valuable resource for modern agriculture.

The studies included in this Research Topic highlight that the functions of endophytic fungi cannot be considered independently of their plant hosts and environmental contexts. The same functional traits may translate into different outcomes depending on host genotype, environmental conditions and interactions with other members of the microbiome. This perspective moves endophyte research beyond the search for individual beneficial strains toward a more integrated understanding of plant–microbe systems and the development of context-dependent microbial consortia.

One of the most compelling aspects of endophytic fungi is their extraordinary diversity. In a study on Inca nut (*Plukenetia volubilis*) (Xu et al.), 168 fungal strains representing 36 genera, including one previously unknown, were isolated from healthy leaves. Within the extracted endophytic communities, the predominance of *Nigrospora, Cercospora*, and *Hypoxylon* reflected local ecological conditions, while *Colletotrichum* and *Pseudopithomyces* were consistently found at all the sampling sites. Many isolates displayed traits associated with plant growth promotion, including siderophore production, phosphate solubilization, IAA synthesis, cellulase and chitinase production. Selected isolates significantly enhanced seed germination and seedling growth in Chinese kale and tomato, underscoring their potential as biofertilizers and biostimulants.

In another study (Zhou et al.), a total of 179 fungal isolates representing 50 taxa were obtained from two Chinese medicinal vines, *Gynostemma pentaphyllum* and *Gynostemma longipes*. Higher species richness and isolation rates characterized the first species. The culture extracts of these fungi displayed varying degrees of antibacterial activity, with a *Fusarium* isolate exhibiting strong broad-spectrum activity.

Another investigation (Dong et al.) focused on understanding how endophytes stimulate plant growth at the molecular level. Working with *Festuca sinensis*, researchers compared endophytic isolates belonging to the genera *Trichoderma, Cladosporium*, and *Penicillium*. A general ability to improve plant biomass, root development and tillering capacity, as well as to enhance stress-tolerance, was observed through distinct mechanisms. In fact, multi-omics analyses showed that *Trichoderma* mainly promoted growth through auxin-related pathways and the activation of genes involved in hormone signaling and disease resistance, whereas *Cladosporium* appeared to stimulate steroid biosynthesis and hormone transport, and *Penicillium* primarily influenced carbon metabolism. These findings demonstrate that different fungal taxa may provide complementary functions, paving the way to designing tailored microbial consortia for specific crops and environmental conditions.

A study on wheat stripe rust caused by *Puccinia striiformis* f. sp. *tritici* (Khan et al.), demonstrated how indigenous endophytic fungi can boost plant immunity. Two isolates identified as *Curvularia lunata* and *Aspergillus fumigatus* significantly improved seed germination, plant growth, chlorophyll and flavonoid contents, and biomass production in susceptible wheat cultivars. Moreover, treated plants exhibited enhanced antioxidant defenses, reduced lipid peroxidation, and lower disease severity. The study suggests that these endophytes can simultaneously contribute to plant vigor and disease resistance, through the production of bioactive metabolites, resistance-related proteins, and signaling molecules such as salicylic acid and jasmonic acid. Such mechanisms mirror the complex immune priming processes increasingly recognized as central features of plant-microbe mutualisms.

Among the most attractive applications of endophytes is their role as biological control agents. Indeed, the increasing regulatory limitations on chemical pesticides call for alternative solutions in plant disease management. In this context, a study on hazelnut (*Corylus avellana*) (Nicoletti et al.) explored the antibacterial properties of endophytic fungi against two economically important bacterial pathogens, *Pseudomonas avellanae* and *Xanthomonas arboricola* pv. *corylina*. Screening 50 fungal isolates obtained from hazelnut tissues led to the selection of four active strains, belonging to *Cladosporium perangustum, Talaromyces purpureogenus, Nemania diffusa*, and the *Hypoxylon fuscum* species complex. Their culture filtrates suppressed bacterial growth by approximately 90% *in vitro*, supporting the assumption that endophytic fungi may participate in defensive mutualism.

Another study carried out on hazelnut (Brugneti et al.) disclosed taxonomic implications, after the finding that isolates formerly identified as *Fusarium lateritium* should instead be assigned to the *Fusarium citricola* species complex. Through phylogenetic analyses and species-delimitation algorithms, the authors pointed out that current taxonomic boundaries within this complex may require revision. However, the relevance of this study extends beyond taxonomy. Indeed, many *Fusarium* species present an ambiguous ecological position, functioning at times as pathogens and at other times as endophytes. Accurate identification is therefore essential not only for biodiversity studies but also to distinguish beneficial symbionts from potential threats, and to evaluate risks associated with mycotoxin production.

Taken together, these studies reveal several recurring themes. First, endophytic fungi represent an immense reservoir of biodiversity whose agricultural value is only beginning to be explored. Second, their beneficial effects may arise through multiple mechanisms, including nutrient acquisition, phytohormone production, stimulation of plant metabolism, enhancement of stress tolerance, and direct antagonism against pathogens. Third, advances in molecular biology, genomics, transcriptomics, and metabolomics are providing unprecedented insights into the functional basis of these interactions, making it increasingly possible to move from empirical observations to predictable applications.

The broader significance of this Research Topic lies in its alignment with the goals of sustainable agriculture. Climate change, emerging diseases, soil degradation, and the need to reduce dependence on chemical inputs are driving the search for environmentally friendly technologies. Endophytic fungi offer a particularly promising solution because they work through naturally evolved biological relationships. Unlike external microbial inoculants, endophytes occupy internal plant tissues, potentially providing greater persistence and more intimate interactions with their hosts. The challenge ahead is to translate laboratory and greenhouse findings into reliable field applications. Factors such as host specificity, environmental variability, microbial competition, and formulation technologies must still be addressed. Nevertheless, the evidence emerging from recent studies strongly suggests that endophytic fungi have the potential to become genuine agricultural allies, supporting crop productivity, strengthening resistance to diseases, and contributing to the transition toward more sustainable and resilient farming systems.

